# A Non-invasive Digital Biomarker for the Detection of Rest Disturbances in the SOD1G93A Mouse Model of ALS

**DOI:** 10.3389/fnins.2020.00896

**Published:** 2020-09-01

**Authors:** Elisabetta Golini, Mara Rigamonti, Fabio Iannello, Carla De Rosa, Ferdinando Scavizzi, Marcello Raspa, Silvia Mandillo

**Affiliations:** ^1^Institute of Biochemistry and Cell Biology-National Research Council (IBBC-CNR), CNR-Campus International Development (EMMA-INFRAFRONTIER-IMPC), Monterotondo, Italy; ^2^Tecniplast SpA, Buguggiate, Italy

**Keywords:** ALS, SOD1G93A, sleep, home cage monitoring, DVC^®^, mouse behavioral phenotyping, circadian rhythm, locomotion

## Abstract

Amyotrophic Lateral Sclerosis (ALS) is a devastating neurodegenerative disease that affects both central and peripheral nervous system, leading to the degeneration of motor neurons, which eventually results in muscle atrophy, paralysis, and death. Sleep disturbances are common in patients with ALS, leading to even further deteriorated quality of life. Investigating methods to potentially assess sleep and rest disturbances in animal models of ALS is thus of crucial interest. We used an automated home cage monitoring system (DVC^®^) to capture irregular activity patterns that can potentially be associated with sleep and rest disturbances and thus to the progression of ALS in the SOD1G93A mouse model. DVC^®^ enables non-intrusive 24/7 long term animal activity monitoring, which we assessed together with body weight decline and neuromuscular function deterioration measured by grid hanging and grip strength tests in male and female mice from 7 until 24 weeks of age. We show that as the ALS progresses over time in SOD1G93A mice, activity patterns start becoming irregular, especially during day time, with frequent activity bouts that are neither observed in control mice nor in SOD1G93A at a younger age. The increasing irregularities of activity pattern are quantitatively captured by designing a novel digital biomarker, referred to as Regularity Disruption Index (RDI). We show that RDI is a robust measure capable of detecting home cage activity patterns that could be related to rest/sleep-related disturbances during the disease progression. Moreover, the RDI rise during the early symptomatic stage parallels grid hanging and body weight decline. The non-intrusive long-term continuous monitoring of animal activity enabled by DVC^®^ has been instrumental in discovering novel activity patterns potentially correlated, once validated, with sleep and rest disturbances in the SOD1G93A mouse model of the ALS disease.

## Introduction

Amyotrophic Lateral Sclerosis (ALS) is a devastating neurodegenerative disease that affects both central and peripheral nervous system, and it is characterized by the degeneration of upper and lower motor neurons that will result in muscle atrophy, paralysis and death within 2–5 years after diagnosis (Kiernan et al., 2011). Death usually comes from respiratory failure. Along with progressive voluntary skeletal muscle weakness and atrophy, symptoms include dysphagia, dysarthria, respiratory dysfunction and sleep disturbances. Sleep disruption is very common in ALS and it is often related to hypoventilation, hypoxia, hypercapnia, restless legs, immobilization, nocturnal cramps, and pain (Lo Coco et al., 2011; Boentert, 2019). Sleep disturbances are also very common in other neurodegenerative diseases, as Alzheimer’s, Parkinson’s and have an enormous impact on the quality of life of patients (Iranzo, 2016; Falup-Pecurariu and Diaconu, 2017; Winer et al., 2019). Circadian and sleep dysfunctions are often premorbid and can serve as early diagnostic markers of neurodegeneration.

Some studies in animal models of neurodegenerative diseases tackle this important issue (Vanderheyden et al., 2018; Medeiros et al., 2019) but few are specific for ALS models. It is therefore crucial to provide evidence of this early symptom and to propose a suitable tool to investigate circadian and sleep related disruption in a classical mouse model of ALS as the SOD1G93A transgenic strain (Gurney et al., 1994). The SOD1G93A mouse model we used expresses multiple copies of the human mutated form of the SOD1 gene (on a C57BL/6J background) and it recapitulates the progressive symptomatology of the disease starting around 14–16 weeks of age: muscle weakness, tremors, body weight loss, limb paralysis, respiratory failure and death around 160–170 days or 24–25 weeks of age and it also presents ALS characteristic neurobiological substrates (cortical and spinal cord motor neuron degeneration, gliosis, neuroinflammation) (Leitner et al., 2009; Mina et al., 2018).

To study activity and rest in the SOD1G93A mouse model during the pre-symptomatic and symptomatic stages of the disease we adopted a technology capable of monitoring animal activity directly in the home cage (Richardson, 2015; Bains et al., 2018; Pernold et al., 2019). Such systems are generally designed with the aim of collecting animal activity data 24/7, without interfering with nor handling the animals. This has the potential to unveil animal behaviors occurring at any time during the day (Richardson, 2015) and over extended observation periods ranging from days to months and years (Pernold et al., 2019). The application of 24/7 long term home cage animal monitoring is particularly promising in the field of neurodegenerative diseases, especially the ones that potentially manifest complex modifications of the activity behaviors as the disease progresses over time, and that can be potentially hard to capture when observing animals outside the home cage as in conventional testing procedures (e.g., open field, rotarod, grid hanging, etc.). In this experiment we monitored animals for several months, using the home cage monitoring system, referred to as Tecniplast DVC^®^ (Iannello, 2019), which enables automated and non-intrusive long term data collection.

The main goal of this paper is to explore and better understand disease progression over time while keeping animals in a familiar environment (i.e., the home cage) for most of their life. This may lead to unveil activity patterns that would be hardly measurable otherwise. Particularly, we developed a new digital biomarker to potentially detect rest disturbances by observing animals for extended periods. We considered non-activity as a measure of rest, similar to Pack et al. (2007) in which video-based locomotion has been correlated to EEG measurements to monitor rest and sleep. In addition to home cage monitoring, we also performed tests commonly used in assessing ALS-related symptoms, such as body weight loss and neuromuscular decline (grid hanging, grip strength tests, and monitoring of splay reflex). We demonstrated that the use of home cage monitoring allowed us to capture rest-related disturbances at the symptomatic stage with a peak in good correlation with other symptoms of the disease.

## Materials and Methods

### Subjects

Male B6.Cg-Tg(SOD1^∗^G93A)1Gur/J mice carrying a high copy number of mutant human SOD1 allele (Gurney et al., 1994) were purchased at the Jackson Laboratories (Bar Harbor, ME, United States, stock n. 004435) and a mouse colony was established in-house at the CNR-EMMA-Infrafrontier-IMPC facility (Monterotondo, Italy) by crossing hemizygous transgenic males with C57BL/6J females. Progeny was genotyped by standard PCR following the Jackson Lab protocol^1^. Litter- and sex-matched pups were raised group-housed in standard cages (Thoren, Hazleton, PA, United States) enriched with a transparent red polycarbonate igloo house (Datesand, Manchester, United Kingdom) and with wood shavings contained in single cellulose bags, used as nest paper material (Scobis Uno bags, Mucedola, Settimo Milanese, Italy). Food (Standard Diet 4RF21, Mucedola, Italy) and water were available *ad libitum* on the top of the cage. Room temperature was 21 ± 2°C, relative humidity was 50–60%, and mice were kept in a 12 h light/dark cycle with lights on at 07:00 am until 07:00 pm. Animals were subjected to experimental protocols approved by the Local Animal Welfare Committee and the Veterinary Department of the Italian Ministry of Health (Aut. #914/2016-PR), and experiments were conducted according to the ethical and safety rules and guidelines for the use of animals in biomedical research provided by the relevant Italian laws and European Union’s directives (Italian Legislative Decree 26/2014 and 2010/63/EU). All adequate measures were taken to minimize animal pain or discomfort. Extra wet food was provided inside the cage when the animals were showing a body weight loss of approximately 10%.

### Experimental Design

Male and female wild-type (WT) and transgenic (SOD1G93A) littermate mice at the age of 7 weeks were transferred to the DVC^®^ rack (see below), housing two mice per cage of same sex and genotype and assigned to the following experimental groups: (1) Males, WT *n* = 18 (9 cages); (2) Males, SOD1G93A (TG) *n* = 18 (9 cages); (3) Females, WT *n* = 22 (11 cages); (4) Females, SOD1G93A (TG) *n* = 20 (10 cages). Experiments have been conducted testing three separate cohorts of mice (Cohort I *N* = 18, Cohort II *N* = 30, Cohort III *N* = 30), coming from three breeding generations.

From the age of 7 weeks, all mice were weighed weekly and tested for neuromuscular function using the grid hanging (weekly) and grip strength (every 2 weeks only for the cohort II and III) tests. Hind limbs splay reflex was monitored weekly from the age of 8 weeks to determine disease onset and progression. Grip strength and splay reflex results are presented in the Supporting Information. Mice of the first cohort were sacrificed at 22 weeks of age based on failure on the grid hanging test. Mice of the second and third cohort were monitored until humane endpoint (body weight loss > 20% or loss of righting reflex), around age 24 weeks, and thus sacrificed according to current laws and regulations. Cages were changed every 2 weeks for cohort I (due to mouse facility management priorities) and once a week for cohort II and III, we verified that this difference did not affect data outcome.

### Home Cage Activity Monitoring: Digital Ventilated Cage (DVC^®^) System

All mice were housed in a Digital Ventilated Cage (DVC^®^) rack, which is equipped with a home cage monitoring system capable of automatically measuring animal activity 24/7 (Iannello, 2019). DVC^®^ rack is installed on a standard IVC rack (Tecniplast DGM500, Buguggiate, Italy) by adding sensing technologies externally to the cage, so that neither modifications nor intrusion occur in the home cage environment. Animal locomotion activity is monitored via a capacitance sensing technology by means of 12 contactless electrodes, uniformly distributed underneath the cage floor, which record animal movements based on their presence in each electrode surrounding. In this work, animal activity is captured, similarly, to the activation density metric defined in a previous study (Iannello, 2019), and by choosing to aggregate measurements in bins of 1 min (raw activity data). For simplicity, we refer to this metric as activity throughout the paper. Since this study lasted several months (compared to the 1-min binning), we decided to condense the 1-min raw activity into two distinct measurements per week: (i) average activity during night time (i.e., by averaging all 1-min bins within night period in each week); (ii) average activity during day time. Furthermore, we also investigated the average activity pattern across the entire day (24 h) by observing the minute-based activity. We considered the minute-based activity time series (1440 min across 24 h), smoothed it with a moving average of 60 min and normalized it to peak activity so that the maximum value is one. We then considered the average of this smoothed and normalized curve across all days of the period of interest and for all the cages for each group. Finally, we also analyzed the least active consecutive hour, which may relate to a longer period of rest. The least active hour, for each cage and for each day, is determined by selecting the 60 consecutive min with the lowest average activity across that day (24 h). We found that all least active hours always lie within day time, as shown in the histogram in Supplementary Figure S1.

### Regularity Disruption Index (RDI)

In this paper, we introduce the use of a novel digital biomarker, referred to as Regularity Disruption Index (RDI), which has been developed to capture irregular animal activity patterns. To quantitatively capture these patterns, we designed RDI based on the sample entropy (Richman and Moorman, 2000) as the core metric, in which we set parameters *m* = 2 and *r* = 0.2 (as per notation in Richman and Moorman, 2000), which has been used in some studies to analyze locomotor activity data (Hauge et al., 2011). We computed the sample entropy of the minute activity during day time (i.e., *N* = 720 being the sequence length, that is, the number of minutes in 12 h). Before computing the sample entropy of the time series, we set activity below a given threshold (λ = 0.005) to zero and then applied a Butterworth band-pass filter (independently for each day), whose parameters have been derived with Python library SciPy v.1.1.0. We used a filter of the fourth order and with normalized cut-off frequencies (f_*low*_ = 1/2000 and f_*high*_ = 1/300). We practically observed that band-pass filtration enables more stable results than that without filtering. As for the DVC^®^ activity (see above), we considered the weekly average RDI throughout the paper. We used the same approach to compute RDI during night time (*N* = 720). Similar to the minute-based 24 h activity pattern (with 1-min granularity), we considered the 24 h pattern of RDI. Here, we computed a central moving RDI with a window of 60 min: for each minute *t*, moving-RDI is obtained by computing RDI of the minute activity during the 1 h interval centered in *t* (*N* = 60). Finally, we also computed RDI within the least active consecutive hour (*N* = 60), for each cage and day.

It is worth to mention that RDI is a metric that measures irregularities of a time series (home cage activity in this paper), and it is not influenced by the absolute amount of the activity itself. As an example, a period of activity (divided in 1 min bins) in which all minutes have similar activity levels, gives an RDI approaching 0. Furthermore, RDI is scale invariant: if we multiply the activity levels by a scalar value, RDI does not change. Instead, when minutes of activity are very different with each other, then RDI tends to be large, regardless of the average activity in the period of interest.

### Neuromuscular Function and Body Weight Assessments

#### Grid Hanging Test

Mice were tested weekly from the age of 7 weeks. Each mouse was placed in the center of a wire grid (1-cm squares) raised about 50 cm from a bench covered with sawdust bags. After gentle shaking, the grid was rotated upside down and the mouse was left hanging on it. The latency to fall from the grid was recorded over two trials of 60 s each, with an inter-trial interval of approximately 30 min (Olivan et al., 2015). For each time point (age in weeks) the sum of the two trials was used for the analysis. When the total latency of two trials was under 10 s the test was terminated for that mouse.

#### Grip Strength Test

Mice of the second and third cohort were tested on the grip strength meter apparatus (Bioseb, France) every two weeks from the age of 7 weeks until 21 weeks. Each mouse was held gently by the base of its tail over the top of the grid with its torso in a horizontal position, then it was pulled back steadily until it could no longer resist the increasing force and the grip was released (Mandillo et al., 2008). The grip strength meter digitally displays the maximum force applied as the peak tension (in grams) once the grasp is released. For each mouse the grip test consisted of three trials with forelimbs and three trials with all four paws with an inter-trial interval of 60 s. For each time point (age in weeks) the mean of the three trials was taken as an index of forelimb and all four paws grip strength. Results of four paws measures in this test are in Supporting Information.

#### Body Weight, Splay Reflex, Disease Symptoms, and Humane Endpoint Assessment

Body weight (BW) was measured weekly from age 7 weeks and after each behavioral test. Data were expressed as percentage variation of weight compared to the first measure taken at 7 weeks of age. Hind limbs splay reflex was scored weekly in transgenic mice from the age of 8 weeks. Each mouse was lifted by the tail for 2 s and a score from 0 (full hind limbs extension) to 4 (absence of righting reflex, humane endpoint) were assigned based on the degree of legs extension from the body’s lateral midline (Leitner et al., 2009; Deitch et al., 2014). Symptoms like tremors, loss of splay reflex, BW decline, delayed righting reflex were daily monitored at the late stage of the disease and used to determine the humane endpoint (BW loss > 20%, complete loss of righting reflex).

### Statistical Analysis

Since the same subjects were assessed over time, we performed non-parametric repeated measures analysis on most datasets with Genotype and Sex as between-subject factors and Age (weeks) as within-subject factor (see Pernold et al., 2019). We used the rank-based analysis of variance-type statistic (ATS), as implemented in the nparLD R Software package (Noguchi et al., 2012). We performed the statistical tests until age 20 weeks because after that time some subjects were missing. We ran *post-hoc* analysis where possible, using two-sample *T*-tests. Since the conventional Bonferroni correction is too conservative for strongly correlated repeated measures (in our case measurements are taken quite frequently with respect to the time scale of development of the disease), we used the D/AP procedure to correct tests for multiple comparisons (Sankoh et al., 1997). This method considers correlation between outcomes and is equivalent to no correction for perfectly correlated measures and to Bonferroni correction for completely independent measures. For reference, we reported tables with different correction methods (no correction, Bonferroni correction, D/AP correction) in the Supporting Information. We performed *post-hoc* analysis for both DVC^®^ and conventional measures until the age of 20 weeks in which all the cages or mice were still present. In all other cases presented in the paper, not involving repeated measures, we performed two-sample *T*-test.

We used Python to process and visualize data and R to run all statistics (version 3.4.3), with significance level α = 0.05. For the analysis of DVC^®^ metrics (activity and RDI) the statistical unit is the cage, while for the analysis of physical assessments (BW, grid hanging test, grip strength test, splay reflex score) the statistical unit is the individual mouse. Days of cage changing were excluded from the analysis. Moreover, since a male WT mouse died during the experiment at week 16, we excluded its cage from the analysis of DVC^®^ metrics. Data are generally presented as mean ± SEM (relatively to cages for DVC^®^ metrics and to mice for tests outside the cage).

## Results

### DVC^®^-Based Activity and RDI Patterns

**FIGURE 1:**
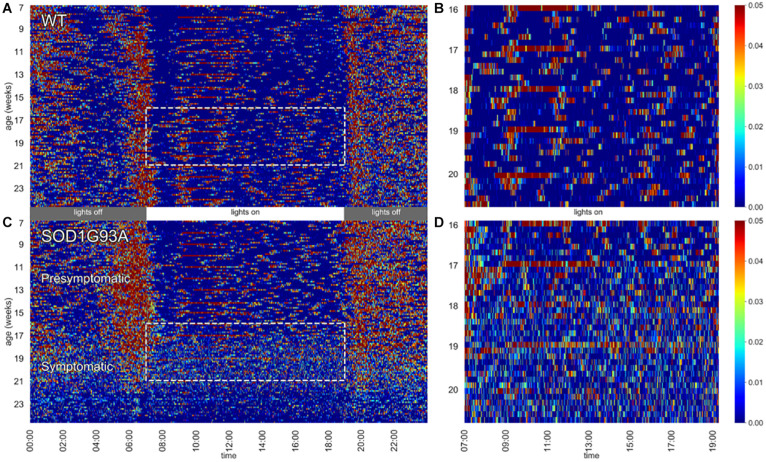
Heat maps of activity. Left panels depict 24 h activity across 7–24 weeks of age in a cage of WT **(A)** and a cage of SOD1G93A **(C)** male mice (*n* = 2 per cage). Dotted boxes represent activity during day time between age 16–20 weeks, which are then zoomed in the respective right panels **(B,D)**. Weekly cage change related peaks of activity appear clearly as red stripes.

**FIGURE 2:**
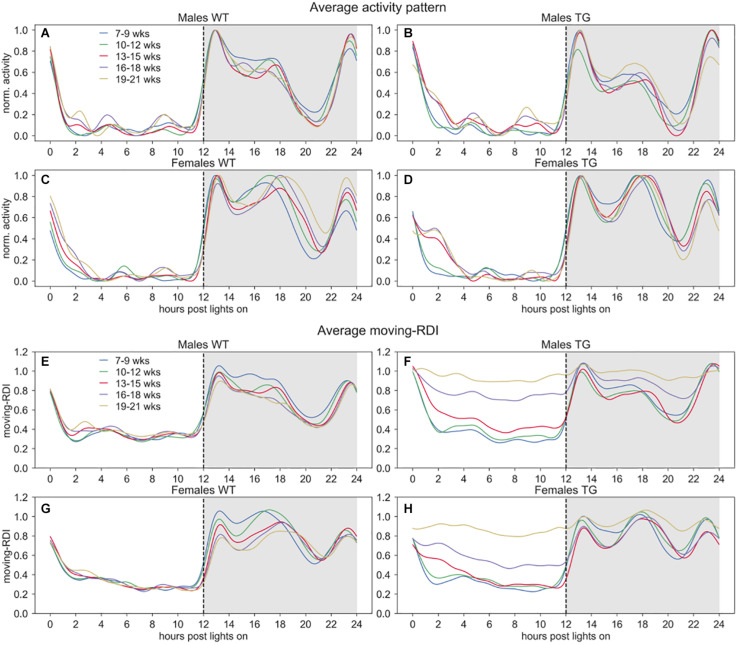
Activity and moving-RDI patterns over 24 h. Panels **(A–D)** show the minute-based activity time series over 24 h, smoothed with a moving average of 60 min and normalized to peak activity (=1.0), then averaged across cages of the same group and weeks of the same 3-weeks period. Panels **(E–H)** show the central moving RDI computed on the minute-based activity with a sliding window of 60 min, then smoothed with a moving average of 60 min and averaged across cages of the same group and weeks of the same 3-weeks period. Lights-on at 07:00 AM (hour 0), lights-off at 07:00 PM (hour 12).

The DVC^®^ system monitored night and day time activity of mice 24/7 for about 4 months. As expected, mice are more active during the night period (Pernold et al., 2019) particularly closer to light/dark transitions (Figures 1, 2).

In Figure 1 heat maps of two representative cages of male wild-type (WT) and SOD1G93A (TG) mice (2 mice per cage) show how the activity is distributed across the 24 h (Figures 1A,C) along the experiment (from age 7 until 24 weeks of age). While the activity of WT mice is very stable across the whole experiment, we observed a clear reduction of nocturnal activity in SOD1G93A cages around 19 weeks of age, corresponding to the fully symptomatic stage in this mutant strain (tremors, hind limb weakness, overt weight loss). Interestingly, from around 16 weeks of age we observed an atypical pattern of day time activity in the SOD1G93A cages compared to WT littermates (highlighted with the dotted boxes in Figures 1A,C). This pattern is also visible during the night time hours when the mice show lower activity (4–2 h before lights-on). In Figures 1B,D we zoomed in the day time activity of mice to better appreciate the difference in the activity pattern in a period in which mice usually rest. In Figure 1B the peaks of activity (in red) during routine weekly cage changes are evident.

To qualitatively observe these activity-based patterns for all the cages, we show the average time series of normalized activity and moving-RDI over 24 h in Figure 2. As expected, the overall activity is higher during night time and interestingly we observed qualitative differences in the peaks of activity between males and females. All groups show very low activity during day time and interestingly also during some hours of the night, right before the response to lights-on (hours 20–22), suggesting that mice may have a resting stage during the lights-off phase, too.

In Figure 1, we observed that TG mice (developing ALS) start showing activity patterns that become irregular, with frequent activity bouts, which are not seen in control groups or TG mice at a younger age. The activity anomalies observed especially in the diurnal pattern of activity in SOD1G93A cages led us to develop RDI as a digital biomarker to quantitatively measure the irregularities of these patterns. To qualitatively observe how this index changes during the 24 h, we first show the average moving-RDI time series for each group and over time (Figures 2E–H). The pattern of moving-RDI of WT mice and TG mice at a younger age is similar to the activity pattern, with low values during the most inactive hours and peaks corresponding to peaks of activity (when activity is very variable on a minute-by-minute basis). In both male and female TG mice (Figures 2F,H), we show that the average curves arise with increasing age. The increase is remarkable during day time: although the average activity remains very low (Figures 2B–D), the moving-RDI index becomes as high as the periods with high activity (e.g., peaks during the night), showing that it is the activity pattern that changed, i.e., minutes become largely variable between each other, and thus irregular, and not the amount of activity, which remains substantially unchanged. The increase of the TG curves is also visible during night time, especially during periods in which the average activity is lower than the peaks.

After qualitatively observing activity and RDI over 24 h, we quantitatively summarized these metrics in the following sections.

### DVC^®^ Monitoring of Day and Night Activity

**FIGURE 3:**
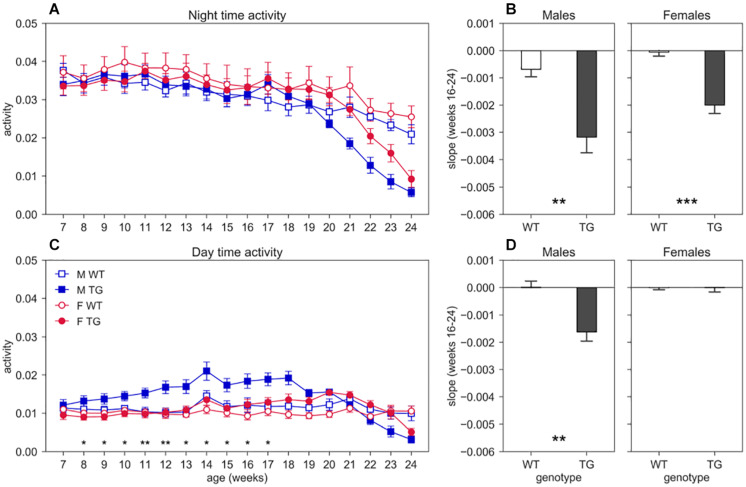
Night and day time activity. Average weekly activity (±SEM) over night **(A)** and day **(C)** time across 7–24 weeks of age in male (M, blue squares) and female (F, red circles) cages of WT (open symbols) and SOD1G93A (TG, filled symbols) mice. N of cages per group: M WT = 8; M TG = 9; F WT = 11; F TG = 10. We found no significant D/AP *post-hoc* test (until week 20) when comparing day activity between WT and TG with both males and females. Since we found a significant Genotype X Sex X Age interaction for day time activity (nparLD test, Genotype X Sex X Age interaction: Statistic = 2.356, *df* = 4.161, *p* < 0.05) we compared TG males with TG females with D/AP *post-hoc* procedure in C (*p < 0.05, **p < 0.01, black stars). We observed a clear decreasing trend of day and night activity in TG mice compared to WT, but we could not perform tests after week 20, since some cages were missing. Panel **(B)** shows the slope of the linear regression computed for the weekly night time activity points in weeks of age 16–24, while panel **(D)** shows the results for day time activity. (**p < 0.01, ***p < 0.001 two sample *T*-test).

We show the average weekly cage activity during night (Figure 3A) and day (Figure 3C) time over the course of the experiment (age 7–24 weeks). We observe a decreasing trend of both day and night activity in TG mice compared to WT, that however, is not statistically significant for night activity since we had to limit the analysis up to week 20 to have all the animals for all the weeks, due to repeated measures testing that require all subjects at all time points. For day activity, we instead found a significant interaction between genotype and age (nparLD test, Genotype × Age interaction: Statistic = 6.415, *df* = 4.161, *p* < 0.001). To further investigate the trend of activity in TG mice, starting at age 16–17 weeks and continuing even after the 20th week, we computed the linear regression on the night and day activity between weeks 16 and 24, by considering only weeks with two mice in the cage. The slopes of the regression lines are shown in the bar plot of Figure 3B (night) and Figure 3D (day). As expected, the slope for night activity (Figure 3B) for both TG males and females are considerably larger than those of the control mice (two-sample *T*-test, males: *T* = 3.802, *df* = 15, *p* < 0.01; females: *T* = 5.470, *df* = 19, *p* < 0.001). For day activity, we found a significant difference in the slope only in males (Figure 3D, two-sample *T*-test, males: *T* = 3.876, *df* = 15, *p* < 0.01; females: *T* = 0.233, *df* = 19, *p* > 0.05).

### Regularity Disruption Index (RDI)

**FIGURE 4:**
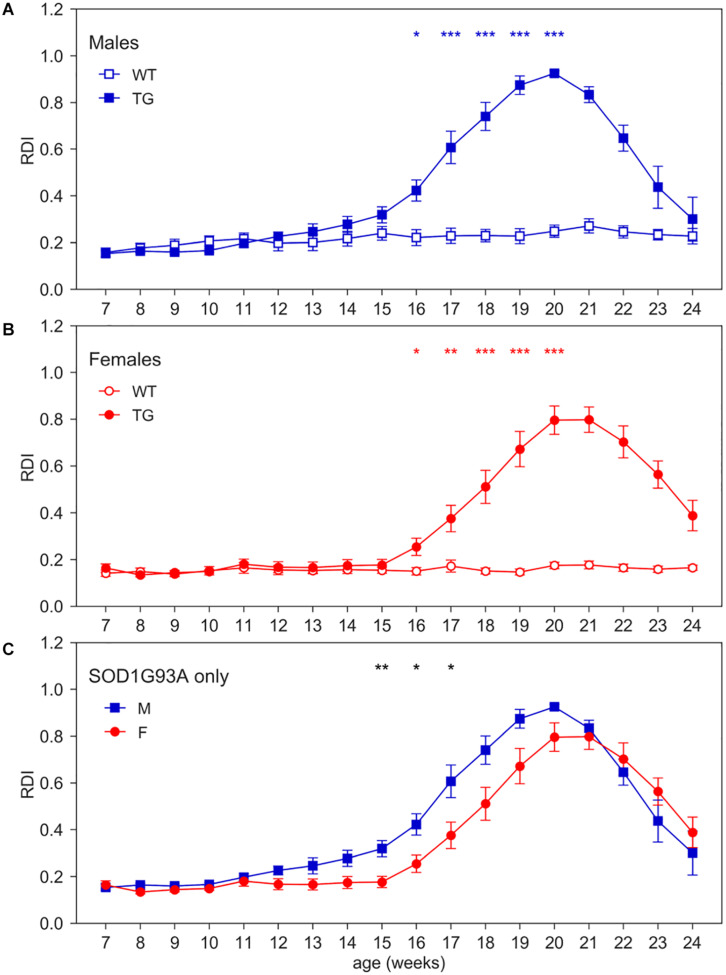
Day-time Regularity Disruption Index (RDI). Average weekly RDI curves (±SEM) across 7–24 weeks of age in **(A)** males, **(B)** females, **(C)** TG males and females measured during day time. N of cages per group: M WT = 8; M TG = 9; F WT = 11; F TG = 10. In males **(A)** and females **(B)** *p < 0.05, **p < 0.01, ***p < 0.001 WT vs. TG, D/AP *post-hoc* procedure. Since we found a significant Genotype X Sex X Age interaction (nparLD test, Genotype X Sex X Age interaction: Statistic = 3.084, *df* = 6.429, *p* < 0.01), we compared TG males with TG females with D/AP *post-hoc* procedure in **(C)** (*p < 0.05, **p < 0.01, black stars).

To quantitatively capture the increase of irregular patterns of activity in SOD1G93A cages over the development of the disease (Figure 1), we computed RDI during the 12 h of day time, when mice are more inactive and where we observed a higher increase of moving-RDI in Figures 2F,H. Figure 4 clearly shows how this index is rapidly increasing over weeks of age and is remarkably high in both male and female TG mice with a peak around 20 weeks of age, while remaining essentially flat in WT mice (nparLD test, Genotype effect: Statistic = 10.054, *df* = 1, *p* < 0.01; Genotype × Age interaction: Statistic = 25.459, *df* = 6.429, *p* < 0.001). Note that RDI curves for TG mice are bell-shaped, meaning that after an increase and a peak, then follows a decline. The reasons need to be further investigated, but it is likely due to the fact that, after the peak, around 20 weeks, TG mice neuromuscular functions deteriorate, and their ability to move is severely impaired. Consequently, the chance to be inactive increases, and thus the RDI decreases. The average RDI curves for TG males and TG females show similar but shifted patterns (Figure 4C). We thus computed the cross-correlation (Lewis, 1995) between the two curves and found that they are maximally similar (i.e., maximum of the cross-correlation) when the curve of TG females is anticipated by 1 week. However, onset of RDI, defined as the age at which TG mice differ significantly from WT (D/AP *Post-hoc* test), is 16 weeks both in males and females.

**FIGURE 5:**
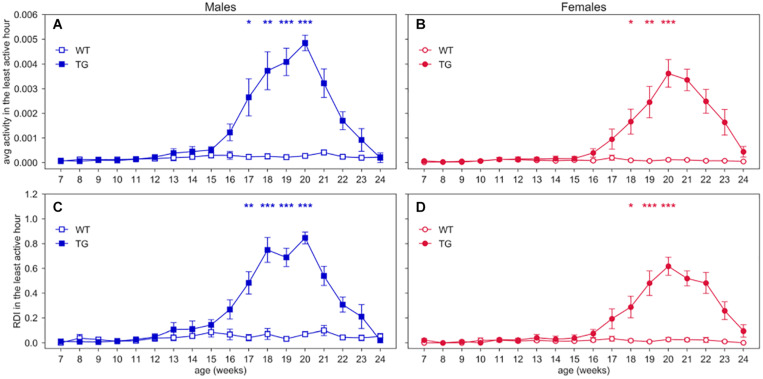
Activity and RDI within the least active hour. Average weekly activity and RDI curves (±SEM) across 7–24 weeks of age in **(A,C)** males, **(B,D)** females, measured during the least active hour of the day. N of cages per group: M WT = 8; M TG = 9; F WT = 11; F TG = 10. In males **(A,C)** and females **(B,D)** *p < 0.05, **p < 0.01, ***p < 0.001 WT vs. TG, D/AP *post-hoc* procedure.

To better observe RDI during potential resting periods, we also calculated RDI within the least active consecutive hour (Figure 5), which may relate to a long period of rest. In this time window, we found that both activity and RDI in TG mice increase over weeks, with a peak at around 20 weeks of age (nparLD test for activity, Genotype × Age interaction: Statistic = 18.928, *df* = 7.542, *p* < 0.001; nparLD test for RDI, Genotype × Age interaction: Statistic = 13.909, *df* = 7.053, *p* < 0.001). Particularly, Figures 5A,B show that there is always a consecutive hour of the day when the activity is approximately zero for WT mice, as opposed to TG mice. The least active hour is found always during the lights-on period (Supplementary Figure S1). The scatter plots in Supplementary Figure S2 show the direct relationship between the average weekly activity and RDI in the least consecutive hour, for all the cages.

Since we observed an increase of moving-RDI also during night time (Figure 2), we also analyzed the night time RDI (Supplementary Figure S3). This index is higher than day time RDI for WT mice and TG mice at young ages, because resting periods are less frequent and thus activity is more irregular during lights-off hours. Still, we observe an increase of night-time RDI for TG mice, that becomes significantly higher than WT mice (see Supplementary Material).

### Classical ALS Model-Related Measures: Neuromuscular Function and Body Weight Assessments

**FIGURE 6:**
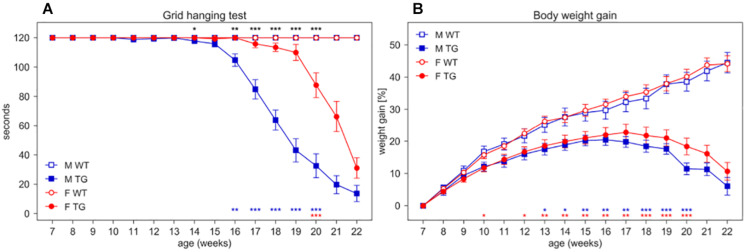
Grid hanging test and body weight gain. **(A)** Latency to fall per mouse (average time ± SEM) of mice hanging from an inverted grid. **(B)** Average percentage of body weight gain ± SEM (per mouse) calculated from initial weight at age 7 weeks. Measures were taken weekly (weeks 7–22) in male (M, blue squares) and female (F, red circles) WT (open symbols) and SOD1G93A (TG, filled symbols) mice. N of mice per group: M WT = 17; M TG = 18; F WT = 22; F TG = 20. In each sex group (males = blue stars, females = red stars) *p < 0.05, **p < 0.01, ***p < 0.001 WT vs. TG, D/AP *post-hoc* procedure. Since we found a significant Genotype X Sex X Age interaction in the grid hanging test (nparLD test, Genotype X Sex X Age interaction: Statistic = 11.630, *df* = 5.206, *p* < 0.001) we compared TG males with TG females with D/AP *post-hoc* tests in A (*p < 0.05, **p < 0.01, ***p < 0.001, black stars).

Beside the deficit in body weight gain, SOD1G93A mice show the expected decline in neuromuscular function assessed by grid hanging and grip strength tests and splay reflex score (Figure 6 and Supplementary Figures S4, S5).

In the grid hanging test (Figure 6A), the nparLD analysis revealed a significant effect of Genotype, Sex and Age factors and all their interactions (*p* < 0.001). In particular, male TG mice show a significant reduction in grid hanging duration compared to WT starting at 16 weeks of age (D/AP *Post-hoc* tests) while female TG mice show a reduction starting much later (20 weeks of age).

We expressed body weight as percentage of weight gain calculated from initial weight at 7 weeks of age (Figure 6B). While WT mice show a constant weight gain over time, TG mice show a slower weight gain since early ages (nparLD test from week 8–20, Genotype factor: Statistic = 33.778, *df* = 1, *p* < 0.001; Genotype × Age interaction: Statistic = 39.300, *df* = 4.408, *p* < 0.001). Female TG mice weigh consistently and significantly less than WT starting at week 12 while TG male weigh significantly less than WT starting from week 13 (Figure 6B).

We measured grip strength only in mice of the second and third cohort (*N* = 59) and, as expected, TG mice showed a decrease in grip strength over time, and a significant difference between WT and TG mice at early ages (9 weeks of age for males and 11 weeks of ages for females, as in Supplementary Figure S4). We also observed in SOD1G93A mice the loss of hind limbs splay reflex at the age of 14–15 weeks (Score = 1; Supplementary Figure S5), the score increased with age implying a progressive severity of this neurological deficit, worse in males than in females.

### Effects of Husbandry and Experimental Procedures on Activity and RDI

**FIGURE 7:**
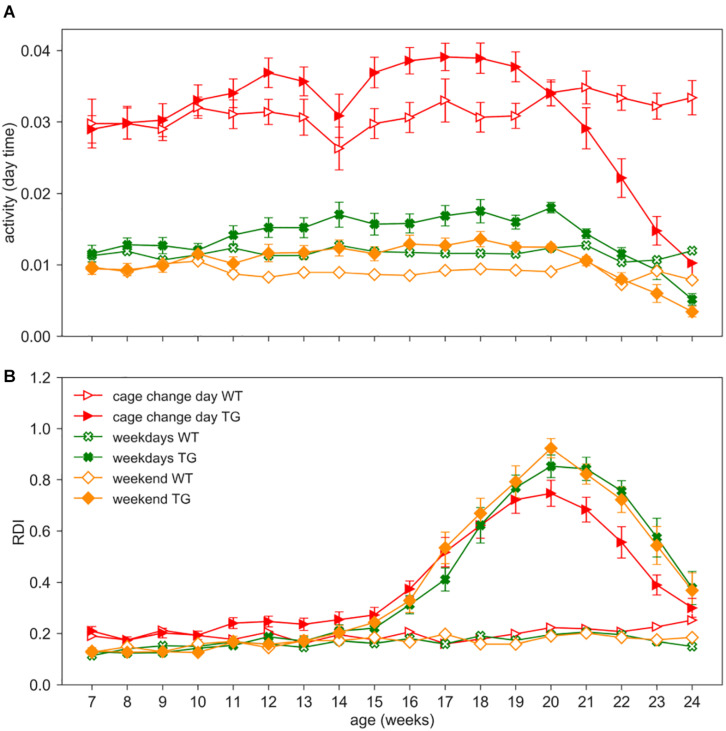
Impact of procedures. Day time activity **(A)** and RDI **(B)** curves across weeks 7–24 in WT (open symbols) and TG (filled symbols) (males and females) during cage change days (red triangles), weekdays (green crosses, 2 days in which procedures as grid test or body weight assessment were performed) or weekends (orange diamonds, Saturday and Sunday, without procedures). Data are mean ± SEM. N of cages per group: WT = 19; TG = 19. No differences were observed in RDI between weekdays and weekends in both genotypes. RDI is not influenced by personnel activity in the room nor by experimental procedures.

As an additional investigation, we assessed the impact of procedures such as cage change, grid and grip tests, and body weight measurements on activity and RDI metrics. We separated the average activity and RDI measured during weekends (when no procedures are performed) and weekdays in which either cage change or experimental procedures are performed (cage change day or weekdays) (Figure 7).

We performed the nparLD test with Genotype as between-subject factors and Age (weeks) and Day_type (weekdays or weekend) as within-subject factor. Cage change days as a within-subject factor could not be included in the statistical analysis due to different periodicity in the two cohorts (cage change every two weeks vs. weekly). As expected, day time activity on weekdays is higher than weekends (Figure 7A; nparLD test, Day_type factor: Statistic = 212.245, *df* = 1, *p* < 0.001). Day time activity is also strongly impacted by cage change procedures, as already observed in Pernold et al. (2019), and clearly visible in Figure 7A. For this reason, we remind that days of cage changing were excluded from all the previous analyses. Interestingly, RDI does not show a significant difference between weekdays and weekends (nparLD factor, Day_type factor: Statistic = 1.690, *df* = 1, *p* > 0.05; Day_type X Genotype interaction: Statistic = 0.034, *df* = 1, *p* > 0.05), and similarly, the RDI is not substantially affected during cage change days (Figure 7B).

## Discussion

In this paper, we presented a novel digital biomarker with the aim to provide a non-invasive method for the identification of ALS-related symptoms in male and female SOD1G93A mice. We monitored animals in their home cage via the DVC^®^ system, which is capable of non-intrusively detecting animal locomotor activity 24/7. We considered previously developed metric such as DVC^®^ activity (Iannello, 2019; Pernold et al., 2019) and developed a new metric, referred to as Regularity Disruption Index (RDI), which is designed to capture irregularities in the activity pattern of mice. We also collected measurements on the grid hanging test, grip strength test, hind limbs splay reflex and body weight, which are classically used to monitor the progression of the disease (Knippenberg et al., 2010; Deitch et al., 2014; Olivan et al., 2015).

We showed that RDI is a digital biomarker capable of detecting ALS-related symptoms, similarly, to the decline of neurological and motor functions. The peculiarity of RDI is that it is derived from the raw home-cage activity, thus avoiding handling animals compared to other commonly used procedures such as the grid hanging or the grip strength tests. Moreover, RDI is a robust measure that it is not substantially impacted by cage changing or other procedures performed during day time.

The fully symptomatic stage in which most of the disease related phenotypes are represented can be positioned in the time window 16–22 weeks of age. In this critical interval the RDI-related significant difference WT vs. TG is in fairly good alignment/correspondence with the decline of night time activity, neuromuscular functions, body weight and progressive loss of splay reflex. All these phenotypes appeared earlier in males than females as already reported in other studies (Mccombe and Henderson, 2010). In summary, we thus confirm the phenotypes, onsets and the expected sex differences observed in previous studies, by monitoring SOD1G93A mice using both DVC^®^ activity and classical measures.

We observed that the rise of RDI in TG mice is remarkable especially when computed during day time (in which mice, a nocturnal species, are mostly inactive). The increase of irregularity in day activity pattern in TG mice could reflect disturbances in their rest/sleep behavior. To further investigate this potential relation, we show that RDI increases also within the least active hour of the day, in which mice probably rest most of the time (Huber et al., 2000). The activity of TG mice also increases in this time interval, while it remains approximately zero for WT mice, suggesting that WT mice are almost completely inactive and rest at least for an hour, as opposed to TG mice. All these findings suggest that RDI is an additional marker to detect symptoms of the disease automatically and non-invasively and, once validated, could potentially detect ALS-related sleep fragmentation at the symptomatic stage.

In general, we believe that observing animals in the home cage 24/7 provides clear advantages to experimental data collection, as it allows constant animal monitoring, during both resting and active periods, no animal handling and, crucially, metrics are based on algorithms that are objective and replicable across experiments and sites. This differs from tests performed outside the home cage, which require animal handling during animal resting periods, and often measurements are impacted by operator subjectivity. In addition, we showed that RDI is substantially not influenced by external procedures taking place in the animal room during the day.

The RDI metric, that we suggested being able to capture potential rest/sleep disturbances in ALS models, could be useful as a digital biomarker to detect disease-related phenotypes also in other neurodegenerative disease models. Circadian rhythm and sleep disruption are in fact recognized as common symptoms that negatively affect the quality of life in many diseases such as Parkinson’s, Alzheimer’s, schizophrenia (Iranzo, 2016; Falup-Pecurariu and Diaconu, 2017; Winsky-Sommerer et al., 2019) and also ALS (Lo Coco et al., 2011; Boentert, 2019). Often these disturbances have been recognized as premorbid signs of the disease and are also typical in the aging population (Leng et al., 2019).

In ALS patients, sleep disruption is often caused by respiratory dysfunction and nocturnal muscle cramps and fasciculations accompanied by pain (Ahmed et al., 2016; Boentert, 2019).

Very few studies have explored a sleep- or circadian activity-related phenotype in ALS animal models. Sleep fragmentation has been observed in a TDP-43 *Drosophila* model (Estes et al., 2013). A study in FUS mutant rats reported sleep and circadian rhythm abnormalities that precede cognitive deficits (Zhang et al., 2018). In SOD1G93A mice have been observed sleep-related EEG/EMG abnormalities that increase with age (Liu et al., 2015). Additionally, always in SOD1G93A mice, melatonin has been shown to increase survival (Weishaupt et al., 2006) while light-induced disruption of circadian rhythm anticipated disease onset and reduced survival (Huang et al., 2018). Our findings, though not measuring sleep directly, suggest a surge of rest fragmentation in both male and female SOD1G93A mice at the symptomatic stage. Additional investigations will be planned in order to validate the RDI to consider it a true sleep disturbance index, for example by using electroencephalographic measures and video recordings of sleep behavior.

Overall, we believe that digital biomarkers associated with long-term monitoring of animals affected by neurodegenerative diseases, such ALS, can shed light on behavior and activity patterns that are either unknown or not easily available with conventional, non-continuous animal monitoring. Moreover, the advantages become even more evident when these digital biomarkers can be extracted via technologies capable of non-intrusively monitoring animals 24/7 for several weeks and months. We also envision that digital biomarkers such as RDI, can be successfully applied to other diseases where sleep/rest disturbances appear over time, and that they can be used to preliminary assess the efficacy of treatments in large experiments. In fact, systems such as DVC^®^, can be used to monitor hundreds of cages simultaneously and thus would be very helpful for large-scale mouse phenotyping endeavors as the one undertaken for example by the International Mouse Phenotyping Consortium (IMPC) (Meehan et al., 2017; Brown et al., 2018; Joshi et al., 2019).

## Data Availability Statement

All datasets generated for this study are included in the article/Supplementary Material.

## Ethics Statement

The animals were subjected to experimental protocols approved by the Local Animal Welfare Committee and the Veterinary Dept. of the Italian Ministry of Health (Aut. #914/2016-PR), and experiments were conducted according to the ethical and safety rules and guidelines for the use of animals in biomedical research provided by the relevant Italian laws and European Union’s directives (Italian Legislative Decree 26/2014 and 2010/63/EU). All adequate measures were taken to minimize animal pain or discomfort. Extra wet food was provided inside the cage as needed.

## Author Contributions

FI, SM, EG, and MRa: conceptualization. MRi, FI, EG, and SM: data handling, validation, visualization, methodology, and writing – original draft. FI and MRi: formal analysis and software. CD, EG, and SM: investigation. FS: project administration and resources. MRa, FI, and SM: supervision. FI, MRi, SM, and EG: writing – review and editing. All authors contributed to the article and approved the submitted version.

## Conflict of Interest

FI and MRi were employed by Tecniplast SpA, which provided support in the form of salaries for authors FI and MRi. Tecniplast SpA did not have any additional role in the study design, data collection and analysis, decision to publish, or preparation of the manuscript.

The remaining authors declare that the research was conducted in the absence of any commercial or financial relationships that could be construed as a potential conflict of interest.

## Footnotes

1 www.jax.org
