# Supplementary material for: A Non-invasive Digital Biomarker for the Detection of Rest Disturbances in the SOD1G93A Mouse Model of ALS
Source: Front Neurosci. 2020 Sep 1;14:896. doi: 10.3389/fnins.2020.00896 (PMC7490341; doi:10.3389/fnins.2020.00896)
Supplement: Supplementary file 1 [file Table_1.pdf]

Post-hoc analysis	Correction method	week 7	week 8	week 9	week 10	week 11	week 12	week 13	week 14	week 15	week 16	week 17	week 18	week 19	week 20
DAY ACTIVITY (M) *Figure 3C*	*No adjustment*	0.7596	0.7437	0.6881	0.5729	0.4278	0.2993	0.4925	0.2929	0.5473	0.3624	0.1295	0.1174	0.5180	0.9881
	Bonferroni	1.0000	1.0000	1.0000	1.0000	1.0000	1.0000	1.0000	1.0000	1.0000	1.0000	1.0000	1.0000	1.0000	1.0000
	D/AP	0.9857	0.9697	0.9144	0.8830	0.6161	0.4766	0.6828	0.4688	0.7625	0.5439	0.2239	0.2604	0.8995	1.0000
DAY ACTIVITY (F) *Figure 3C*	*No adjustment*	0.5952	0.7503	0.7022	0.5555	0.8953	0.7874	0.9598	0.9134	0.9720	0.7708	0.6053	0.6455	0.8098	0.5337
	Bonferroni	1.0000	1.0000	1.0000	1.0000	1.0000	1.0000	1.0000	1.0000	1.0000	1.0000	1.0000	1.0000	1.0000	1.0000
	D/AP	0.9191	0.9726	0.9324	0.8599	0.9966	0.9456	0.9986	0.9944	0.9983	0.9269	0.8324	0.9410	0.9997	0.9583
DAY ACTIVITY (M vs F TG) *Figure 3C*	*No adjustment*	0.1773	**0.0155**	**0.0102**	**0.0067**	**0.0036**	**0.0034**	**0.0078**	**0.0159**	**0.0111**	**0.0155**	**0.0093**	**0.0239**	0.1570	0.9276
	Bonf.	1.0000	0.2172	0.1433	0.0936	0.0505	**0.0482**	0.1095	0.2232	0.1561	0.2164	0.1300	0.3341	1.0000	1.0000
	D/AP	0.3504	**0.0289**	**0.0181**	**0.0139**	**0.0067**	**0.0060**	**0.0141**	**0.0293**	**0.0198**	**0.0272**	**0.0171**	0.0655	0.5830	1.0000
RDI (M) *Figure 4A*	*No adjustment*	0.8337	0.5225	0.2638	0.0843	0.5116	0.4504	0.3616	0.2112	0.1062	**0.0034**	**0.0003**	**<0.0001**	**<0.0001**	**<0.0001**
	Bonferroni	1.0000	1.0000	1.0000	1.0000	1.0000	1.0000	1.0000	1.0000	1.0000	**0.0473**	**0.0038**	**<0.0001**	**<0.0001**	**<0.0001**
	D/AP	1.0000	0.9715	0.8142	0.3453	0.9699	0.8492	0.7440	0.5194	0.3129	**0.0116**	**0.0010**	**<0.0001**	**<0.0001**	**<0.0001**
RDI (F) *Figure 4B*	*No adjustment*	0.3182	0.3992	0.6873	0.8658	0.6241	0.7080	0.6312	0.5627	0.4096	**0.0155**	**0.0030**	**<0.0001**	**<0.0001**	**<0.0001**
	Bonferroni	1.0000	1.0000	1.0000	1.0000	1.0000	1.0000	1.0000	1.0000	1.0000	0.2176	**0.0425**	**0.0006**	**0.0000**	**0.0000**
	D/AP	0.6975	0.9129	0.9978	0.9999	0.9456	0.9617	0.9514	0.8874	0.7678	**0.0368**	**0.0079**	**0.0002**	**<0.0001**	**<0.0001**
RDI (M vs F TG) *Figure 4C*	*No adjustment*	0.6412	0.0195	0.2430	0.1172	0.5469	0.0682	0.0668	**0.0258**	**0.0032**	**0.0101**	**0.0187**	**0.0262**	**0.0352**	0.0634
	Bonferroni	1.0000	0.2736	1.0000	1.0000	1.0000	0.9543	0.9354	0.3613	0.0450	0.1411	0.2619	0.3673	0.4928	0.8872
	D/AP	0.9993	0.0991	0.7414	0.4518	0.9619	0.1783	0.1844	0.0667	**0.0089**	**0.0258**	**0.0463**	0.0825	0.1369	0.3280
ACTIVITY in the least active hour (M) *Figure 5A*	*No adjustment*	0.6660	0.2123	0.6389	0.3273	0.8852	0.6304	0.4011	0.3518	0.2438	**0.0296**	**0.0090**	**0.0008**	**0.0000**	**0.0000**
	Bonferroni	1.0000	1.0000	1.0000	1.0000	1.0000	1.0000	1.0000	1.0000	1.0000	0.4141	0.1263	**0.0108**	**0.0002**	**0.0000**
	D/AP	1.0000	0.7940	0.9982	0.9463	1.0000	0.9641	0.8289	0.7752	0.6157	0.0942	**0.0290**	**0.0025**	**0.0000**	**0.0000**
ACTIVITY in the least active hour (F) *Figure 5B*	*No adjustment*	0.0912	0.6838	0.3379	0.9355	0.9601	0.8035	0.5839	0.4017	0.5126	0.0774	0.0766	**0.0043**	**0.0009**	**<0.0001**
	Bonferroni	1.0000	1.0000	1.0000	1.0000	1.0000	1.0000	1.0000	1.0000	1.0000	1.0000	1.0000	0.0601	**0.0129**	**<0.0001**
	D/AP	0.2024	0.9990	0.6732	0.9999	1.0000	0.9794	0.8983	0.6889	0.8398	0.1557	0.1538	**0.0113**	**0.0052**	**<0.0001**
RDI in the least active hour (M) *Figure 5C*	*No adjustment*	0.4034	0.3500	0.2675	0.8465	0.5752	0.8089	0.3187	0.4723	0.3253	**0.0443**	**0.0005**	**<0.0001**	**<0.0001**	**<0.0001**
	Bonferroni	1.0000	1.0000	1.0000	1.0000	1.0000	1.0000	1.0000	1.0000	1.0000	0.6200	**0.0066**	**0.0004**	**<0.0001**	**<0.0001**
	D/AP	0.9944	0.9570	0.8791	1.0000	0.9924	0.9998	0.7994	0.9478	0.8075	0.1558	**0.0020**	**0.0001**	**<0.0001**	**<0.0001**
RDI in the least active hour (F) *Figure 5D*	*No adjustment*	0.0586	0.9469	0.3304	0.3409	0.8411	0.6581	0.4363	0.5569	0.3606	0.1269	0.0597	**0.0045**	**0.0001**	**<0.0001**
	Bonferroni	0.8210	1.0000	1.0000	1.0000	1.0000	1.0000	1.0000	1.0000	1.0000	1.0000	0.8362	0.0624	**0.0012**	**<0.0001**
	D/AP	0.2573	1.0000	0.7714	0.9613	0.9996	0.9649	0.8512	0.9233	0.7872	0.3011	0.1683	**0.0145**	**0.0005**	**<0.0001**
GRID TEST (M) *Figure 6A*	*No adjustment*	NaN	NaN	NaN	NaN	0.1283	0.3386	0.5551	**0.0159**	0.0630	**0.0013**	**<0.0001**	**<0.0001**	**<0.0001**	**<0.0001**
	Bonferroni	NaN	NaN	NaN	NaN	1.0000	1.0000	1.0000	0.2225	0.8814	**0.0186**	**0.0001**	**<0.0001**	**<0.0001**	**<0.0001**
	D/AP	NaN	NaN	NaN	NaN	0.5374	0.9580	0.9998	0.0767	0.2533	**0.0041**	**<0.0001**	**<0.0001**	**<0.0001**	**<0.0001**

GRID TEST (F) *Figure 6A*	*No adjustment*	NaN	NaN	NaN	NaN	NaN	NaN	0.3466	NaN	0.3000	0.3000	0.1034	**0.0210**	0.0572	**0.0002**
	Bonferroni	NaN	NaN	NaN	NaN	NaN	NaN	1.0000	NaN	1.0000	1.0000	1.0000	0.2943	0.8015	**0.0032**
	D/AP	NaN	NaN	NaN	NaN	NaN	NaN	0.9950	NaN	0.9886	0.7577	0.2918	0.0686	0.1745	**0.0010**
GRID TEST (M vs F TG) *Figure 6A*	*No adjustment*	NaN	NaN	NaN	NaN	0.0985	0.2981	0.2981	**0.0089**	**0.1196**	**0.0005**	**0.0001**	**<0.0001**	**<0.0001**	**<0.0001**
	Bonferroni	NaN	NaN	NaN	NaN	1.0000	1.0000	1.0000	0.1252	1.0000	**0.0075**	**0.0008**	**<0.0001**	**<0.0001**	**0.0005**
	D/AP	NaN	NaN	NaN	NaN	0.4551	0.9387	0.9706	**0.0464**	0.4946	**0.0017**	**0.0002**	**<0.0001**	**<0.0001**	**0.0001**
WEIGHT GAIN (M) *Figure 6B*	*No adjustment*		0.2655	0.4152	0.0494	0.1404	0.0531	**0.0312**	**0.0159**	**0.0045**	**0.0040**	**0.0009**	**0.0005**	**<0.0001**	**<0.0001**
	Bonferroni		1.0000	1.0000	0.6424	1.0000	0.6905	0.4050	0.2061	0.0589	0.0521	**0.0122**	**0.0063**	**0.0008**	**<0.0001**
	D/AP		0.4889	0.5827	0.0659	0.1955	0.0695	**0.0393**	**0.0198**	**0.0057**	**0.0050**	**0.0012**	**0.0007**	**<0.0001**	**<0.0001**
WEIGHT GAIN (F) *Figure 6B*	*No adjustment*		0.3861	0.1035	**0.0196**	**0.0294**	**0.0083**	**0.0031**	**0.0055**	**0.0027**	**0.0011**	**0.0007**	**0.0003**	**<0.0001**	**<0.0001**
	Bonferroni		1.0000	1.0000	0.2549	0.3824	0.1081	0.0398	0.0719	**0.0349**	**0.0142**	**0.0091**	**0.0039**	**0.0001**	**<0.0001**
	D/AP		0.9517	0.2379	**0.0413**	0.0618	**0.0147**	**0.0051**	**0.0092**	**0.0046**	**0.0019**	**0.0012**	**0.0005**	**<0.0001**	**<0.0001**
