# Supplementary material for: A Non-invasive Digital Biomarker for the Detection of Rest Disturbances in the SOD1G93A Mouse Model of ALS
Source: Front Neurosci. 2020 Sep 1;14:896. doi: 10.3389/fnins.2020.00896 (PMC7490341; doi:10.3389/fnins.2020.00896)
Supplement: Supplementary file 2 [file Table_2.pdf]

Post-hoc analysis	Correction method	week 7	week 8	week 9	week 10	week 11	week 12	week 13	week 14	week 15	week 16	week 17	week 18	week 19	week 20
NIGHT RDI (M)<br>*Figure Suppl. 2*	*No adjustment*	0.0784	0.9150	0.0229	0.1359	0.6577	0.2077	0.1540	0.0746	0.9816	0.1272	**0.0011**	**<0.0001**	**<0.0001**	**<0.0001**
	Bonferroni	1.0000	1.0000	0.3204	1.0000	1.0000	1.0000	1.0000	1.0000	1.0000	1.0000	**0.0149**	**0.0004**	**0.0006**	**0.0001**
	D/AP	0.3663	1.0000	0.1031	0.5913	0.9950	0.6502	0.5448	0.3856	1.0000	0.4486	**0.0053**	**0.0001**	**0.0002**	**<0.0001**
NIGHT RDI (F)<br>*Figure Suppl. 2*	*No adjustment*	**0.0140**	0.5156	0.0744	0.1965	0.0951	0.0627	0.8400	0.1026	0.2044	0.2676	0.2972	0.0624	**0.0016**	**<0.0001**
	Bonferroni	0.1965	1.0000	1.0000	1.0000	1.0000	0.8784	1.0000	1.0000	1.0000	1.0000	1.0000	0.8742	**0.0227**	**0.0006**
	D/AP	0.0504	0.9465	0.2704	0.4981	0.2525	0.1886	0.9942	0.2964	0.4525	0.5575	0.6594	0.2433	**0.0095**	**0.0003**

Post-hoc analysis	Correction method	week 7	week 9	week 11	week 13	week 15	week 17	week 19	week 21
GRIP TEST (M)<br>*Figure Suppl. 3*	*No adjustment*	0.2218	**0.0109**	**<0.0001**	**0.0037**	**0.0408**	**0.0001**	**<0.0001**	**<0.0001**
	Bonferroni	1.0000	0.0871	**0.0002**	**0.0298**	0.3266	**0.0010**	**<0.0001**	**<0.0001**
	D/AP	0.6482	**0.0298**	**0.0001**	**0.0105**	0.1207	**0.0003**	**<0.0001**	**<0.0001**
GRIP TEST (F)<br>*Figure Suppl. 3*	*No adjustment*	0.9382	**0.0339**	**0.0001**	**0.0001**	**<0.0001**	**<0.0001**	**<0.0001**	**<0.0001**
	Bonferroni	1.0000	0.2709	**0.0010**	**0.0009**	**0.0003**	**<0.0001**	**<0.0001**	**<0.0001**
	D/AP	1.0000	0.0820	**0.0003**	**0.0002**	**0.0001**	**<0.0001**	**<0.0001**	**<0.0001**
