# Supplementary material for: A Non-invasive Digital Biomarker for the Detection of Rest Disturbances in the SOD1G93A Mouse Model of ALS
Source: Front Neurosci. 2020 Sep 1;14:896. doi: 10.3389/fnins.2020.00896 (PMC7490341; doi:10.3389/fnins.2020.00896)
Supplement: Supplementary file 3 [file Presentation_1.PDF]

Supplementary Material

1 RDI

In S1 Fig, we showed the histogram of the center of the least active hours. The least active hour, for each cage and for each day, is determined by selecting the 60 consecutive minutes with the lowest average activity across that day (24 hours). As expected, we found that the least active hours always lie within day time. The scatter plots in S2 Fig show the relationship between the average activity and the average RDI in the least active consecutive hour, for each cage and week. TG mice show a contemporary increase of RDI and average activity in this time interval.

In S3 Fig, we observe an increase of night-time RDI over weeks for TG mice, that becomes significantly higher than WT mice (nparLD test, Genotype X Age interaction: Statistic=21.039, df=6.544, $p<0.001$). Onset of night-time RDI is slightly anticipated in males (age 17 weeks) with respect to females (age 18 weeks), as in S3 Fig A and B (D/AP Post-hoc test).

2 Grip-test and hind limbs splay reflex score

Grip strength was measured in mice of the second and third cohort (N=59) and, as expected, TG mice showed a decrease in grip strength over time, starting at 9 and 11 weeks of ages in males and females, respectively (S4 Fig, nparLD test, Genotype factor: Statistic=87.810, df=1, $p<0.001$; Genotype X Age factor: Statistic=19.436, df=5.874, $p<0.001$). A statistically significant difference between WT and TG mice appears in week 9 for males and week 11 for females.

We monitored the hind limb splay reflex weekly from age 8 weeks only in SOD1G93A mice. After 22 weeks data are not shown since some animals reached a score of 4 and were sacrificed. The loss of hind limbs splay reflex started at the age of 14-15 weeks when collapse or partial collapse of leg extension towards lateral midline was observed during tail suspension (Score=1; S5 Fig); the score increased with age implying a progressive severity of this neurological deficit, worse in males than in females.

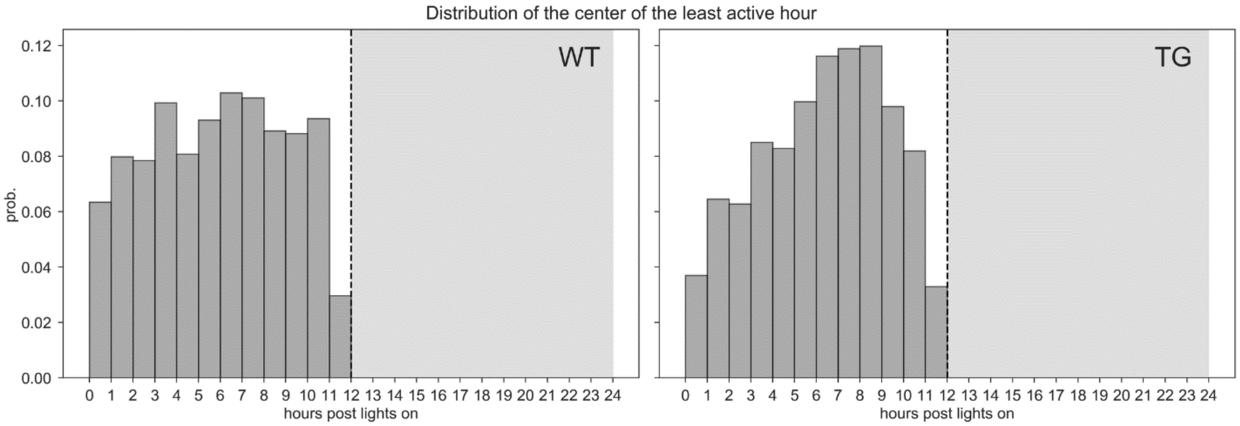

Supplementary Figure 1. Histograms of the center of the least active hour in WT and TG mice. Data of each day and each cage are displayed. All these hours were computed lie within day time. Lights-on at 07:00 AM (hour 0), lights-off at 07:00 PM (hour 12).

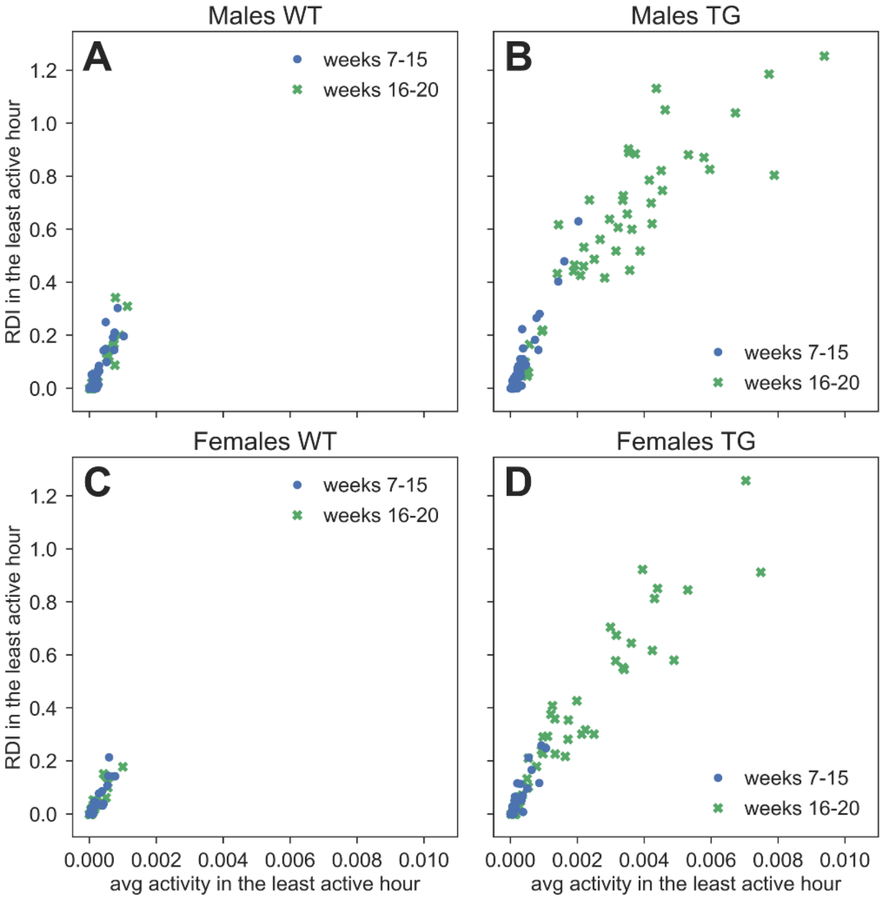

Supplementary Figure 2. Activity and RDI of the least active consecutive hour. Average weekly activity and average weekly RDI for each cage from week 7 to 20. Blue circle points represent weeks from 7 to 15, green crosses represent weeks from 16 to 20, which is the crucial interval where RDI increases (symptomatic stage).

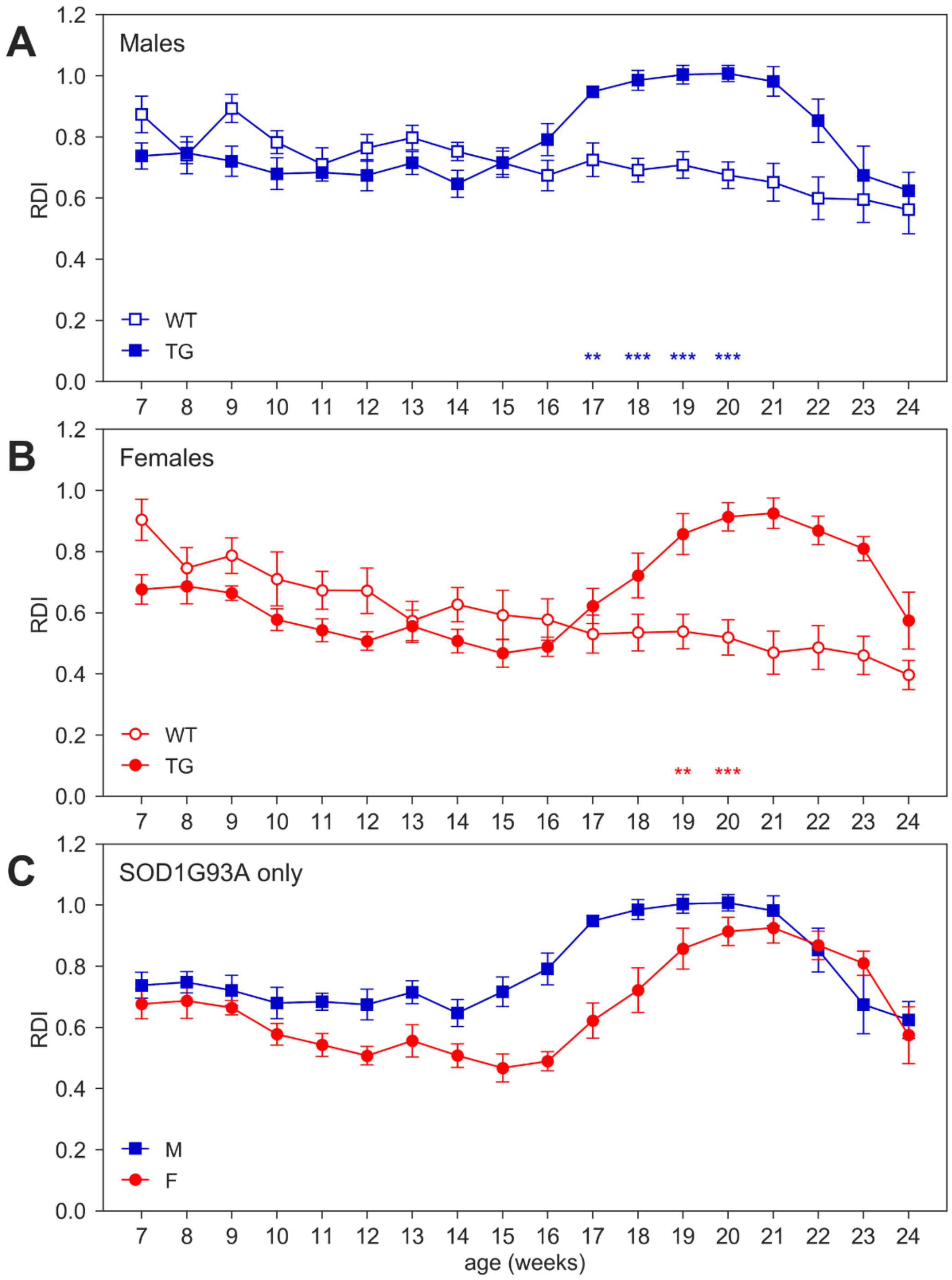

Supplementary Figure 3. Night-time Regularity Disruption Index (RDI). Average RDI curves (± SEM) across 7-24 weeks of age in (A) males, (B) females, (C) TG males and females measured during day time. N of cages per group: M WT=8; M TG=9; F WT=11; F TG=10. In males (A) and females (B), **p<0.01, ***p<0.001 WT vs. TG, D/AP post-hoc procedure.

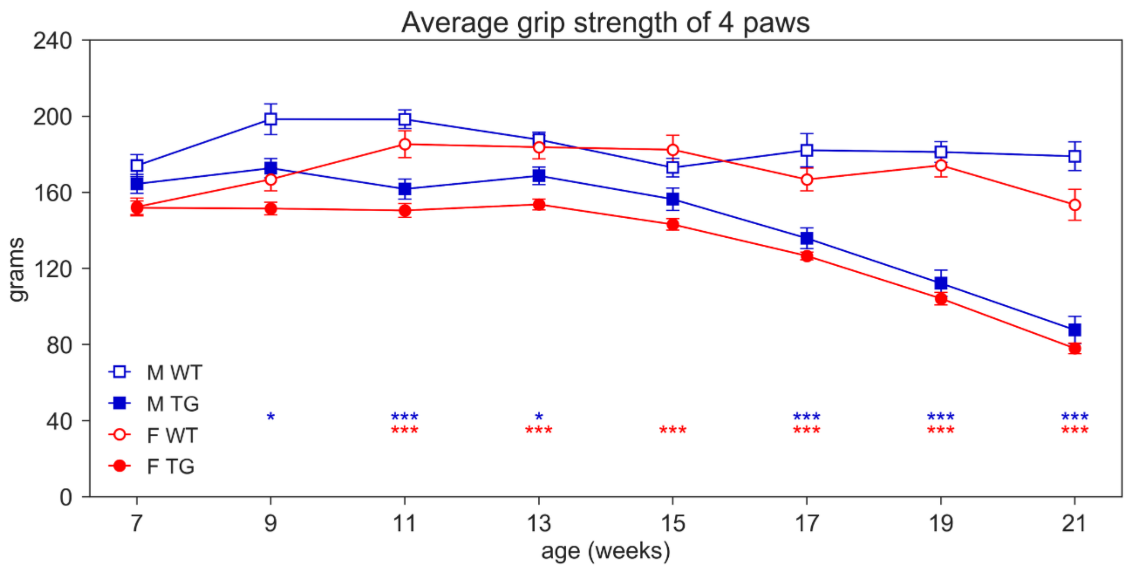

Supplementary Figure 4. Grip strength (average ± SEM) from 7 to 21 weeks of age. Grip strength performance was assessed every two weeks in male (M, blue squares) and female (F, red circles) WT (open symbols) and SOD1G93A (TG, filled symbols) mice of Cohort II and III (N=59). N of mice per group: M WT=13; M TG=14; F WT=16; F TG=16. In each sex group *p<0.05, ***p<0.001 WT vs. TG, D/AP post-hoc procedure.

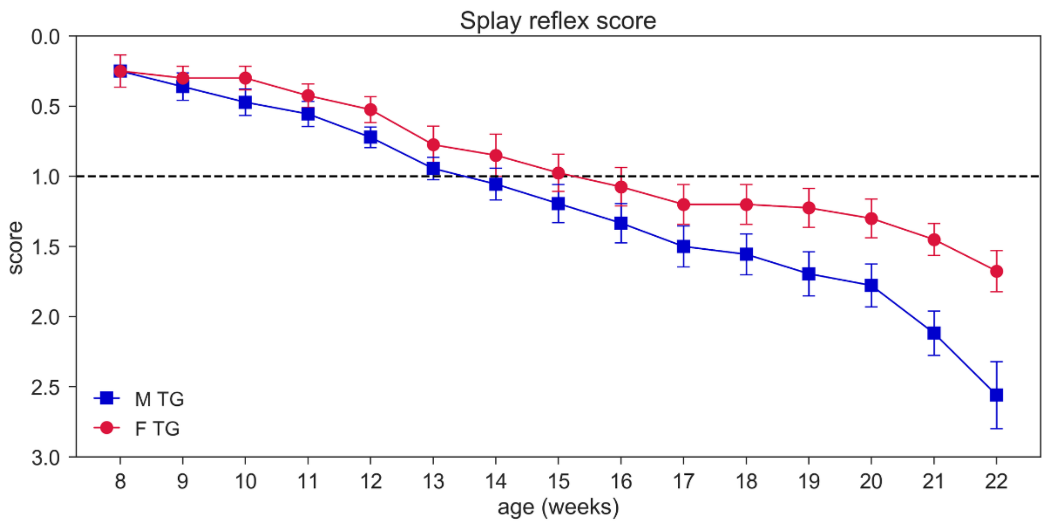

Supplementary Figure 5. Hind limbs splay reflex score (average ± SEM) from 8 to 22 weeks of age in male and female SOD1G93A mice. Dotted line at score 1 indicating initial loss of splay reflex.

frontiers
